# The effect of a 10-week swimming exercise program on emotional state in female sport sciences students

**DOI:** 10.3389/fpsyg.2026.1814833

**Published:** 2026-05-22

**Authors:** Hande Yazıcıoğlu Çalışan, Yağmur Yıldız, Ladislav Batalik, Metin Yüceant

**Affiliations:** 1 Department of Sports and Health, Aksaray University, Aksaray, Türkiye; 2 Department of Physiotherapy and Rehabilitation, Faculty of Medicine, Masaryk University, Brno, Czechia; 3 Department of Rehabilitation, University Hospital Brno, Brno, Czechia; 4 Department of Public Health, Faculty of Medicine, Masaryk University, Brno, Czechia; 5 Rehabilitation Clinic, Faculty of Medicine, Masaryk University, Brno, Czechia; 6 Faculty of Sports Sciences, Aksaray University, Aksaray, Türkiye

**Keywords:** emotional wellbeing, happiness, hopelessness, swimming exercise, university students

## Abstract

University students may experience a range of academic, social, and psychological stressors that can negatively affect emotional wellbeing. Although physical exercise is widely recognized as beneficial for mental health, evidence regarding the emotional effects of structured swimming programs in specific university subgroups remains limited. The aim of this study was to examine the effects of a 10-week structured swimming exercise program on emotional wellbeing, assessed through happiness and hopelessness levels, in female students enrolled in a Faculty of Sport Sciences. This study used an experimental pretest-posttest control group design. A total of 42 female students aged 18–22 years were randomly assigned to a Swimming Exercise Group (*n* = 21) or a Control Group (*n* = 21). The Swimming Exercise Group participated in a supervised swimming program twice per week for 10 weeks, whereas the Control Group maintained their usual routines without any structured exercise intervention. Emotional status was assessed before and after the intervention using the Beck Hopelessness Scale and the Oxford Happiness Questionnaire–Short Form. Data were analyzed using mixed-design ANOVA, and effect sizes were calculated using partial eta squared (η^2^*p*). The results revealed significant group × time interaction effects for both hopelessness and happiness (*p* < 0.05). Hopelessness decreased significantly in the Swimming Exercise Group, whereas no meaningful change was observed in the Control Group (η^2^*p* = 0.30). In contrast, happiness increased significantly only in the Swimming Exercise Group, with a large effect size (η^2^*p* = 0.41). A 10-week structured swimming exercise program was associated with statistically significant improvements in self-reported emotional wellbeing in female sport sciences students. However, these findings should be interpreted within the context of a relatively homogeneous, physically active, and non-clinical sample, and should not be generalized to the broader university student population. Swimming may represent a promising supportive exercise modality in similar student groups, although further research is needed in more diverse university populations.

## Introduction

The university years represent more than a period of academic knowledge acquisition. They constitute a critical transitional phase in which lifestyles are shaped, coping strategies for stress are developed, and psychological resilience is strengthened. During this period, students often experience an intense process of adjustment due to increasing academic demands, concerns about future career prospects, economic pressures, and changes in their social environment. Accordingly, the higher prevalence of stress, mood fluctuations, anxiety, and depressive symptoms among young adults has increased the need for practical approaches that support the emotional wellbeing of university students (Liu et al., 2025; Huang et al., 2024). In this context, physical exercise has emerged as one of the most accessible and preventive strategies.

The positive effects of physical activity on psychological wellbeing have long been recognized. In particular, studies involving university students report significant associations between exercise participation and variables such as mood, life satisfaction, and psychological resilience. Exercise is considered a powerful behavioral tool that supports stress management, reduces symptoms of depression and anxiety, and enhances subjective wellbeing (Huang et al., 2024; Hu et al., 2025). Recent meta-analyses further indicate that physical activity–based interventions generally produce favorable outcomes in mental health indicators among university students (Liu et al., 2025). However, these findings also raise an important question regarding which types of exercise are more effective for specific psychological outcomes.

From this perspective, water-based exercises and swimming have attracted increasing attention due to their distinct physiological and psychological characteristics. Swimming is a whole-body activity that places minimal load on the joints and offers a sustainable exercise option for many individuals. The buoyancy and calming properties of water may reduce perceived exertion during exercise, thereby facilitating adherence. In addition, the rhythmic breathing patterns and repetitive movements involved in swimming may promote a more balanced emotional response.

Studies examining the relationship between aquatic exercise and mental health suggest that water-based activities may exert beneficial effects on mood and anxiety symptoms. A systematic review by Tang et al. (2022) reported that aquatic exercise significantly improves mental health outcomes, particularly when performed at light to moderate intensity. Furthermore, studies conducted with university students indicate that swimming training may have positive effects on negative emotional states (Saatchian, 2024). From a broader perspective, water environments, often referred to as “blue spaces,” have been associated with psychological wellbeing due to their calming and restorative properties (Huang, 2026). Research on open-water swimming has also reported reductions in perceived stress and improvements in mental wellbeing (Burlingham et al., 2022).

Students enrolled in faculties of sport sciences constitute a particularly relevant group for investigating these effects. Although these students generally maintain more physically active lifestyles, they may still be exposed to psychological stressors related to performance expectations, the intensity of practical coursework, and concerns about professional careers. Moreover, a positive attitude toward exercise may facilitate participation in an intervention but does not necessarily guarantee spontaneous emotional improvement. Therefore, examining the effects of a structured exercise program such as swimming on emotional status within an experimental framework may provide important scientific and practical contributions.

Despite the generally positive findings regarding the psychological effects of aquatic exercise, intervention studies conducted with university students using clearly defined durations and experimental–control designs remain limited. Variations in measurement tools and intervention protocols further restrict the generalizability of existing results (Tang et al., 2022; Huang et al., 2024). Accordingly, evaluating the effects of a swimming exercise program implemented over a defined period, such as 10 weeks, may help address this gap in the literature.

In line with this rationale, the aim of the present study was to examine the effects of a 10-week swimming exercise program on emotional status in university students enrolled in a faculty of sport sciences. Emotional status was assessed using both positive (happiness) and negative (hopelessness) dimensions, allowing for a more comprehensive evaluation of the contribution of swimming exercise to students’ psychological wellbeing. The findings are expected to provide a scientific basis for sport-based mental health interventions targeting university students.

## Methods

### Study design

This study was conducted using a quantitative experimental research design with a pretest–posttest control group structure. Experimental designs are widely used to examine the effects of an intervention by comparing outcomes between an intervention group and a control group, and they are considered among the strongest approaches for establishing causal relationships (Büyüköztürk et al., 2020; Creswell and Creswell, 2018).

The primary aim of the study was to investigate the effects of a 10-week structured swimming exercise program on emotional status in university students, focusing on happiness and hopelessness as outcome variables. In line with the experimental framework, participants’ emotional status levels were assessed before the intervention (pretest) and after completion of the 10-week program (posttest). The Swimming Exercise Group participated in the exercise program, whereas the Control Group did not receive any structured exercise intervention during the study period.

The pretest–posttest control group design allowed for the comparison of both within-group changes over time and between-group differences attributable to the intervention. This design also enabled the examination of the interaction between group and time, which was considered the primary indicator of the effectiveness of the swimming exercise program (Karasar, 2021). By employing this controlled experimental approach, the study aimed to strengthen causal inferences regarding the effects of swimming exercise on emotional outcomes.

### Participants

The study population consisted of female university students aged 18–22 years who were enrolled in the Faculty of Sport Sciences at Aksaray University during the 2025–2026 academic year. Participants were recruited through classroom announcements and voluntary enrollment. A total of 42 eligible students who met the inclusion criteria and agreed to participate were included in the study. Written informed consent was obtained from all participants prior to data collection. The final sample consisted of 42 participants, with 21 allocated to the Swimming Exercise Group (experimental) and 21 to the Control Group. Sample size was calculated using G*Power 3.1.9.4, assuming 95% statistical power (1 − *β* = 0.95), a significance level of *α* = 0.05, and a medium effect size (*f* = 0.25). The analysis indicated that at least 18 participants per group were required; therefore, 21 participants were included in each group to account for potential attrition.

The inclusion criteria were as follows: (i) being between 18 and 22 years of age, (ii) being enrolled as a full-time undergraduate student in the Faculty of Sport Sciences, and (iii) not participating in any structured exercise program outside regular academic activities during the study period. The exclusion criteria were: (i) the presence of any chronic cardiovascular, respiratory, or musculoskeletal condition that could limit safe participation in exercise, (ii) current use of psychotropic medication, and (iii) failure to complete either the baseline or post-intervention assessments.

Following eligibility screening and completion of baseline assessments, participants were randomly assigned to either the Swimming Exercise Group (SEG, *n* = 21) or the Control Group (CG, *n* = 21). The allocation sequence was generated by an independent researcher using a computer-based random number generator. This researcher was not involved in participant recruitment, baseline assessment, or intervention delivery. Participant eligibility screening and enrollment were conducted by members of the research team. Group assignments were placed in sealed, opaque envelopes and were disclosed only after all baseline measurements had been completed, in order to ensure allocation concealment and reduce selection bias.

Due to the nature of the exercise intervention, blinding of participants and the supervising instructor was not feasible. In addition, because the primary outcomes were assessed using self-report questionnaires, the study may have been susceptible to expectancy effects and demand characteristics. To reduce procedural bias, all questionnaires were administered under standardized conditions using the same instructions, setting, and assessment order for both groups.

To reduce the influence of potential confounding factors, all participants were instructed not to use ergogenic aids, to maintain their usual sleep routines, and to avoid substantial dietary changes throughout the study period. No dietary intervention was implemented. Participants assigned to the Control Group were asked to maintain their usual physical activity routines and not to initiate any new structured exercise program during the 10-week study period. To monitor compliance with this instruction, control-group participants were contacted weekly and asked whether they had begun any additional structured exercise. No participant reported initiating a new structured exercise program during the intervention period. However, no objective monitoring method, such as accelerometry, activity logs, or a standardized physical activity questionnaire, was used. Therefore, control-group activity monitoring should be interpreted as self-reported and may not have fully captured variation in habitual physical activity.

Participation in the study was entirely voluntary and was not linked to course grades, academic credit, or any compulsory curricular requirement. Participants were informed that they could withdraw from the study at any time without penalty. No participants withdrew during the study period. Attendance in the Swimming Exercise Group was recorded session by session using standardized attendance forms completed immediately after each session by the supervising instructor. According to these records, all participants in the intervention group completed the 20 scheduled exercise sessions. In addition, regular communication was maintained with participants throughout the intervention period, which may have supported continued engagement. Informal feedback obtained during and after the program suggested that participants generally perceived the swimming sessions positively; however, because these impressions were not collected using a standardized instrument, they were not treated as formal study outcomes.

### Study protocol

The study consisted of a Swimming Exercise Group (SEG, *n* = 21) and a Control Group (CG, *n* = 21). Prior to the initiation of the intervention, all participants in both groups completed the Personal Information Form, the Beck Hopelessness Scale (BHS), and the Oxford Happiness Questionnaire–Short Form (OHQ-SF). All instruments were administered under the supervision of the researcher. Standardized instructions were provided to ensure that participants completed the questionnaires individually and comprehensively. The data obtained at this stage were considered baseline (pretest) measurements and were used to determine initial group characteristics and control for potential baseline differences.

Following baseline assessments, the Swimming Exercise Group participated in a structured swimming exercise program for 10 weeks, conducted twice per week (Wednesday and Friday, 10:00–11:30 a.m.). Participants completed a total of 20 exercise sessions, each lasting 90 min. Each session was structured as follows: 10–15 min of standardized warm-up (light-intensity swimming and mobility exercises), 65–70 min of main swimming exercises, and 5–10 min of cool-down (low-intensity swimming and relaxation).

The exercise program was designed according to the principle of progressive overload. During the first 2 weeks, participants trained at low-to-moderate intensity; during weeks 3–6, training intensity was increased to a moderate level; and during weeks 7–10, participants exercised at moderate-to-high intensity. The progression in intensity was achieved by gradually increasing total swimming distance, number of repetitions, and set structure. Training volume was systematically increased over the course of the intervention, taking into account participants’ physical adaptation levels to ensure safe and effective progression.

Exercise intensity was monitored using the Borg Rating of Perceived Exertion (RPE) scale. During weeks 1–2, participants exercised at an RPE of 11–12 (low-to-moderate intensity); during weeks 3–6, intensity was maintained at RPE 12–14 (moderate intensity); and during weeks 7–10, intensity progressed to RPE 14–16 (moderate-to-high intensity). Rest intervals between sets were standardized at 30 s unless otherwise specified in the training protocol. Attendance was recorded at each session using a standardized attendance sheet completed and signed by the supervising instructor immediately after each training session. This procedure ensured accurate monitoring of participation and confirmed full attendance across the intervention period.

The primary aim of the study was to investigate the effects of a 10-week swimming exercise protocol on the emotional state of female university students aged 18–22 years. All assessments and training sessions were conducted at the semi-Olympic swimming pool of Aksaray University. The pool water temperature was maintained between 26 and 28 °C throughout the study period.

Upon completion of the intervention, posttest assessments were conducted for all participants in both the experimental and control groups. The Beck Hopelessness Scale (BHS) and the Oxford Happiness Questionnaire–Short Form (OHQ-SF) were re-administered to obtain follow-up measurements. The effects of the swimming exercise program on emotional state were evaluated by comparing pretest and posttest scores between and within groups.

Throughout the data collection process, participant confidentiality was strictly maintained. All data were anonymized, coded, and used solely for scientific purposes. Participation was voluntary, and participants were clearly informed that they could withdraw from the study at any time without providing a reason.

To improve the reproducibility of the intervention, the main components and progression of the swimming exercise program are presented in Table 1. The program emphasized basic aquatic adaptation and progressive participation in water-based exercise rather than stroke-specific performance training. It included breathing control drills, kicking exercises, water gymnastics, floating and balance tasks, and board-assisted, fins-assisted, freestyle, and backstroke swimming exercises. All participants had sufficient basic swimming competency for safe pool participation at baseline. The sessions were delivered in a standardized group format, with minor instructor-led adjustments based on individual tolerance and technical adaptation. Based on the distance-based components presented in Table 1, the approximate weekly swimming volume increased from 100 m in the early phase to 2,000 m in the final phase, with a cumulative distance of approximately 7,425 m across the 10-week intervention.

**Table 1:** Ten-week structured swimming exercise program with progressive overload and standardized rest intervals (Güney, 2020; Wang, 2025).

Week	Breathing exercises	Kicking drills	Water gymnastics	Floating and balance exercises	Board exercise	Swim Fins	Freestyle	Backstroke
Week 1	2 × 10–30 s rest	4 × 10–30 s rest	2 × 4–30 s rest	–	–	–	–	–
Week 2	2 × 12–30 s rest	6 × 10–30 s rest	2 × 4–30 s rest	–	–	–	4 × 25 m – 30 s rest	–
Week 3	3 × 10–30 s rest	8 × 15–30 s rest	3 × 6–30 s rest	2 × 2 min – 30 s rest	2 × 25 m – 30 s rest	2 × 25 m – 30 s rest	4 × 25 m – 30 s rest	-
Week 4	3 × 12–30 s rest	10 × 20–30 s rest	3 × 6–30 s rest	2 × 3 min – 30 s rest	3 × 25 m – 30 s rest	3 × 25 m – 30 s rest	5 × 25 m – 30 s rest	-
Week 5	3 × 12–30 s rest	10 × 20–30 s rest	3 × 6–30 s rest	2 × 3 min – 30 s rest	4 × 25 m – 30 s rest	4 × 25 m – 30 s rest	6 × 25 m – 30 s rest	4 × 25 m – 30 s rest
Week 6	3 × 15–30 s rest	12 × 20–30 s rest	3 × 8–30 s rest	2 × 4 min – 30 s rest	4 × 25 m – 30 s rest	4 × 25 m – 30 s rest	6 × 25 m – 30 s rest	4 × 25 m – 30 s rest
Week 7	3 × 15–30 s rest	12 × 25–30 s rest	3 × 8–30 s rest	2 × 4 min – 30 s rest	4 × 50 m – 30 s rest	4 × 50 m – 30 s rest	8 × 50 m – 30 s rest	6 × 50 m – 30 s rest
Week 8	3 × 20–30 s rest	14 × 25–30 s rest	4 × 8–30 s rest	2 × 5 min – 30 s rest	4 × 50 m – 30 s rest	4 × 50 m – 30 s rest	8 × 50 m – 30 s rest	6 × 50 m – 30 s rest
Week 9	3 × 20–30 s rest	15 × 25–30 s rest	4 × 8–30 s rest	2 × 5 min – 30 s rest	6 × 50 m – 30 s rest	6 × 50 m – 30 s rest	10 × 75 m – 30 s rest	8 × 50 m – 30 s rest
Week 10	3 × 20–30 s rest	15 × 30–30 s rest	4 × 10–30 s rest	2 × 5 min – 30 s rest	6 × 50 m – 30 s rest	6 × 50 m – 30 s rest	10 × 100 m – 30 s rest	8 × 50 m – 30 s rest

Intensity was progressively increased across weeks according to the principle of progressive overload. Exercise intensity was monitored using the Borg Rating of Perceived Exertion (RPE) scale, ranging from 11 to 12 during weeks 1–2, 12–14 during weeks 3–6, and 14–16 during weeks 7–10. Rest intervals between sets were standardized at 30 s unless otherwise specified. All sessions were supervised by a certified swimming instructor.

### Data collection tools

#### Personal information form

The Personal Information Form, developed by the researchers, was designed to determine the age of the participants (“How old are you?”). This variable was collected at baseline (pretest) to examine whether there was a statistically significant difference between the groups before the intervention and to demonstrate the overall comparability of the experimental and control groups.

#### Beck Hopelessness Scale

The Beck Hopelessness Scale (BHS) was used to assess participants’ levels of negative expectations and hopelessness about the future. The scale was originally developed by Beck et al. (1974), and its Turkish validity and reliability were established by Durak (1994). The BHS consists of 20 items scored dichotomously (0–1), yielding a total score ranging from 0 to 20, with higher scores indicating greater levels of hopelessness. The scale evaluates future-oriented thoughts across three subdimensions: emotional, motivational, and cognitive hopelessness.

In the present study, the BHS was administered as both a pretest and a posttest to examine changes in hopelessness levels following the 10-week swimming exercise program. Given previous evidence indicating that regular physical exercise may reduce hopelessness, the BHS was considered an appropriate instrument for evaluating negative emotional outcomes in this study.

#### Oxford Happiness Questionnaire–Short Form

The Oxford Happiness Questionnaire–Short Form (OHQ-SF) was used to assess participants’ overall happiness and subjective wellbeing. The scale was developed by Hills and Argyle (2002), and its Turkish adaptation was conducted by Doğan and Akıncı Çötok (2011). The OHQ-SF consists of eight items rated on a 5-point Likert scale (1 = strongly disagree to 5 = strongly agree), with higher total scores indicating higher levels of happiness.

In this study, the OHQ-SF was administered before and after the intervention to evaluate changes in positive affect and subjective wellbeing associated with participation in the swimming exercise program. Considering the potential effects of swimming on physical relaxation, stress reduction, and emotional balance, the OHQ-SF was deemed an appropriate tool for assessing positive emotional changes.

### Statistical analyses

Statistical analyses were performed using IBM SPSS Statistics. Descriptive statistics were calculated for all variables and are presented as mean ± standard deviation. Prior to inferential analyses, the normality of data distribution was assessed using the Shapiro–Wilk test. The results indicated that the data were normally distributed (*p* > 0.05), allowing the use of parametric statistical tests.

Baseline differences between the Swimming Exercise Group and the Control Group were examined using independent samples *t*-tests. To evaluate the effects of the 10-week swimming exercise program on emotional status, a 2 (Time: pretest vs. posttest) × 2 (Group: Swimming Exercise Group vs. Control Group) mixed-design repeated measures analysis of variance (ANOVA) was conducted for each dependent variable. This approach allowed for the examination of the main effects of time and group, as well as the group × time interaction, which was considered the primary indicator of intervention effectiveness.

When significant interaction effects were detected, *post hoc* comparisons were performed using paired-samples *t*-tests to identify within-group changes over time. Effect sizes were calculated using partial eta squared (η^2^*p*) and interpreted according to Cohen (1988) guidelines (small = 0.01, moderate = 0.06, large ≥ 0.14). Effect sizes were reported to provide additional information regarding the magnitude of the observed effects.

### Ethics approval and consent

This study was conducted in accordance with the principles of the Declaration of Helsinki. Ethical approval for the study was obtained from the Ethics Committee of Aksaray University (Decision No: 2026/31; Protocol No: SAGETİK 2026–19; Date: 05.02.2026). Prior to participation, all students were informed in detail about the purpose, procedures, potential risks, and benefits of the study. Written informed consent was obtained from all participants before data collection.

Participation in the study was entirely voluntary, and participants were informed that they could withdraw from the study at any time without providing any justification and without any negative consequences. Confidentiality of participant information was strictly maintained throughout the study. All data were anonymized, securely stored, and used solely for scientific research purposes.

## Results

The study sample consisted of 42 participants: 21 in the Swimming Exercise Group and 21 in the Control Group. The age range of the Swimming Exercise Group was 18–22 years (*M* = 19.76 ± 1.22), whereas the Control Group ranged from 19 to 21 years (*M* = 20.14 ± 0.72). Baseline (pretest) characteristics of participants by groups are given in Table 2.

**Table 2:** Baseline (pretest) characteristics of participants by group.

Variable	Swimming Exercise Group (*n* = 21)	Control Group (*n* = 21)	*t* (df = 40)	p*	Mean difference (95% CI)
Age (years)	19.76 ± 1.22	20.14 ± 0.72	−1.23	0.226	−0.38 [−1.01, 0.25]
BHS^1^	7.57 ± 4.65	5.33 ± 4.49	1.58	0.121	2.24 [−0.61, 5.09]
OHQ-SF^2^	22.76 ± 5.59	25.71 ± 6.01	−1.65	0.107	−2.95 [−6.57, 0.67]

^1^Beck Hopelessness Scale, ^2^Oxford Happiness Questionnaire-Short Form.

As presented in Table 2, no statistically significant differences were found between the groups at baseline (pretest) in terms of age (*p* = 0.226), BHS (*p* = 0.121), and OHQ-SF (*p* = 0.107) according to independent samples *t*-tests results. Moreover, the 95% confidence intervals for mean differences included zero in all cases, further supporting the absence of baseline differences. These findings suggest that the groups were broadly comparable prior to the intervention.

Table 3 shows the distribution and reliability statistics of the scores for the scales used in the study. It was observed that the Skewness - Kurtosis values for the Beck Hopelessness Scale and the Oxford Happiness Scale were within the range of −1.5 to +1.5, and that the data met the assumption of normal distribution. Tabachnick and Fidell (2007) stated that normal distribution can be assumed when Skewness - Kurtosis values are between −1.5 and +1.5. Although skewness and kurtosis values indicated that the data were normally distributed, Levene’s test revealed violations of homogeneity of variance at posttest for both study variables. For hopelessness (BHS), the assumption was satisfied at baseline [*F*(1, 40) = 0.710, *p* = 0.405], whereas a statistically significant violation was observed at posttest [*F*(1, 40) = 6.126, *p* = 0.018]. Similarly, for happiness (OHQ-SF), homogeneity of variance was met at baseline [*F*(1, 40) = 0.549, *p* = 0.463], but a significant violation emerged at posttest [*F*(1, 40) = 7.675, *p* = 0.008]. Given the balanced group design and the robustness of mixed-design ANOVA to moderate violations of this assumption, the analyses were retained as planned, and the results were interpreted with appropriate caution.

**Table 3:** Distribution characteristics and internal consistency coefficients of the study scales.

Scale	Group	Test	n	Skewness	Kurtosis	Cronbach’s alpha
BHS	Swimming Exercise Group	pretest	21	1.094	0.104	0.901
		posttest	21	0.744	0.375	0.768
	Control Group	pretest	21	1.332	1.274	0.874
		posttest	21	0.840	−0.082	0.811
OHQ-SF	Swimming Exercise Group	pretest	21	0.657	−0.035	0.770
		posttest	21	−0.667	1.177	0.708
	Control Group	pretest	21	0.071	−1.046	0.830
		posttest	21	0.175	−1.237	0.806

In addition, it was determined that the Cronbach’s Alpha coefficients obtained from the scales were generally above 0.70. Alpha coefficients of 0.70 and above indicate an acceptable level of internal consistency (Nunnally and Bernstein, 1994). These findings show that the measurement tools used in the study provided reliable data. Reporting the alpha coefficients separately for each measurement time (pretest - posttest) was preferred in order to assess the possible change in internal consistency over time.

Table 4 presents the mean and standard deviation values of the pretest and posttest scores for the levels of hopelessness and happiness among participants in the Swimming Exercise Group and Control Group. A total of 42 participants were included in both measurements, with 21 in the Swimming Exercise Group and 21 in the Control Group.

**Table 4:** Mean and standard deviation values for the pretest and posttest scores of the Swimming Exercise Group and Control Group.

Variable	Group	Test	*M*	SD
BHS	Swimming Exercise Group (*n* = 21)	Pretest	7.57	4.65
		Posttest	3.81	2.22
	Control Group(*n* = 21)	Pretest	5.33	4.49
		Posttest	5.66	3.99
OHQ-SF	Swimming Exercise Group (*n* = 21)	Pretest	22.76	5.59
		Posttest	28.47	3.76
	Control Group (*n* = 21)	Pretest	25.71	6.01
		Posttest	25.76	5.84

M, Mean; SD, Standard deviation.

The change in hopelessness levels based on the pretest and posttest scores of the Swimming Exercise Group and Control Group was examined using a 2 (Time) × 2 (Group) mixed-design repeated measures ANOVA, and the results are presented in Table 5. Partial eta-squared (η^2^p) was used to estimate effect sizes, and 95% confidence intervals (95% CI) are reported.

**Table 5:** Results of a 2 (Time) × 2 (Group) mixed-design repeated measures ANOVA for hopelessness.

Variable	Effect	*F*	df	*p*	η^2^*p*	95% CI (η^2^*p*)
BHS	Time	12.02	1, 40	0.001	0.23	[0.06, 0.40]
	Group	0.02	1, 40	0.866	0.01	[0.00, 0.05]
	Time × Group	17.16	1, 40	0.001	0.30	[0.11, 0.46]

Repeated measures ANOVA results showed that the time × group interaction was statistically significant in terms of hopelessness *F*(1, 40) = 17.16, *p* = 0.001, η^2^*p* = 0.30, 95% CI (0.11, 0.46). This finding suggests that the change in hopelessness levels between the Swimming Exercise Group and Control Group differed over time. Bonferroni-adjusted pairwise comparisons indicated that hopelessness scores significantly decreased in the Swimming Exercise Group from pretest (*M* = 7.57, SD = 4.65) to posttest (*M* = 3.81, SD = 2.22), mean difference = −3.76, 95% CI (−5.17, −2.34), *p* < 0.001. In contrast, the Control Group showed no significant change (pretest: *M* = 5.33, SD = 4.49; posttest: *M* = 5.66, SD = 3.99), mean difference = 0.33, 95% CI (−1.08, 1.75), *p* = 0.636.

The analysis also found the main effect of time to be statistically significant [*F*(1, 40) = 12.02, *p* = 0.001, η^2^*p* = 0.23]. However, given the significant time × group interaction, the main effect of time was evaluated contextually and not interpreted in isolation. The main effect of group was found to be insignificant [*F*(1, 40) = 0.02, *p* = 0.866, η^2^*p* = 0.01].

**Figure 1:**
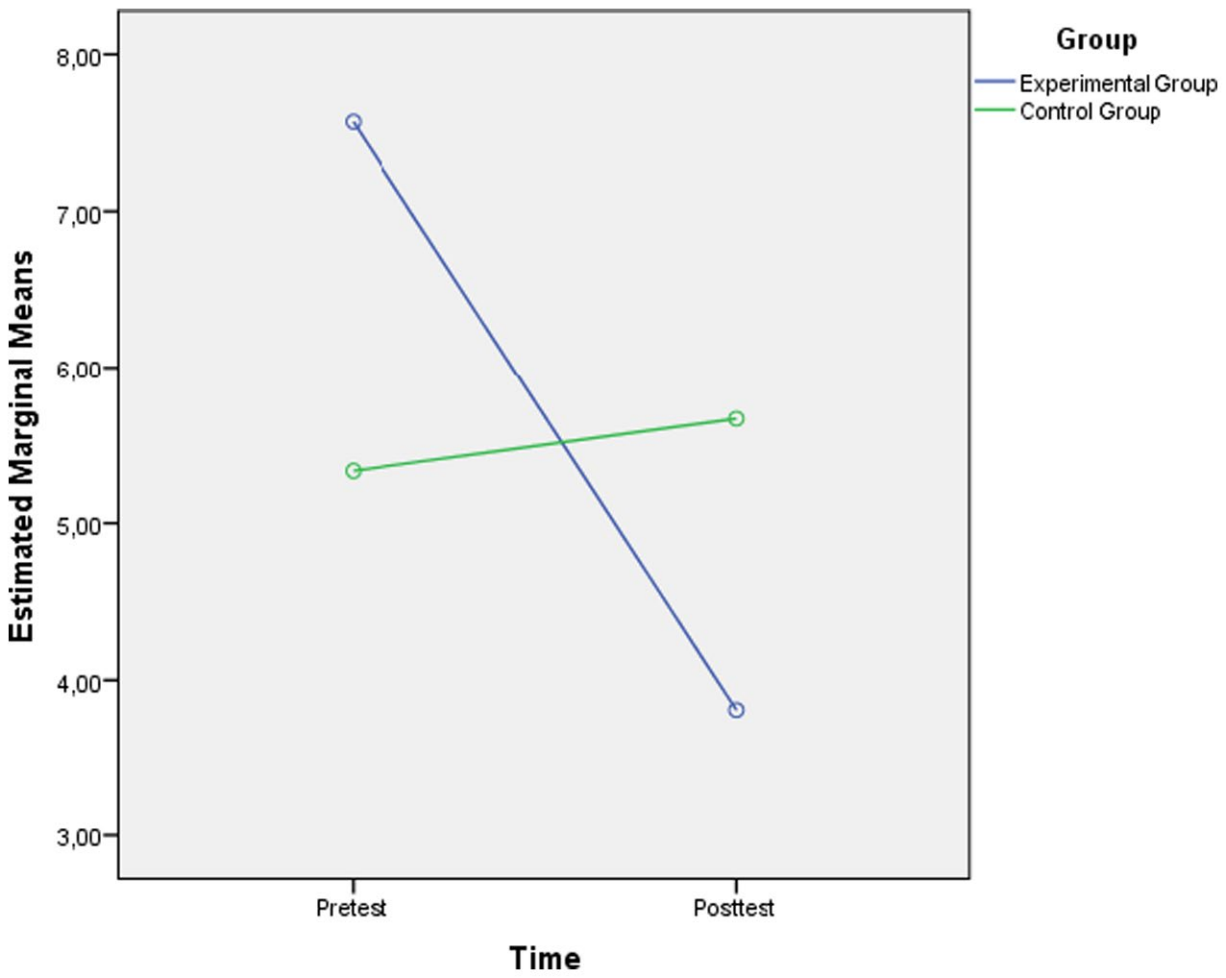
Pretest and posttest hopelessness scores in the Swimming Exercise Group (experimental group) and Control Group.

The graph showing the pretest and posttest mean scores of the swimming exercise group and control group regarding their levels of hopelessness is presented in Figure 1.

As shown in Figure 1, the level of hopelessness in the swimming exercise group decreased significantly from the pretest to the posttest. In the control group, however, no change was observed between measurements.

The change in happiness levels based on the pretest and posttest scores of the Swimming Exercise Group and Control Group was examined using a 2 (Time) × 2 (Group) mixed-design repeated measures ANOVA. The results obtained are presented in Table 6. Partial eta-squared (η^2^p) was used to estimate effect sizes, and 95% confidence intervals (95% CI) were reported where applicable.

**Table 6:** Results of a 2 (Time) × 2 (Group) mixed-design repeated measures ANOVA for happiness.

Variable	Effect	*F*	df	*p*	η^2^*p*	95% CI (η^2^*p*)
OHQ-SF	Time	29.84	1, 40	0.001	0.42	[0.22, 0.56]
	Group	0.06	1, 40	0.940	0.01	[0.00, 0.06]
	Time × Group	28.86	1, 40	0.001	0.41	[0.21, 0.55]

The analysis results indicate that the interaction between time and group is statistically significant in terms of happiness *F*(1, 40) = 28.86, *p* = 0.001, η^2^*p* = 0.41, 95% CI (0.21, 0.55). This interaction suggests that the change in happiness levels over time differed between the Swimming Exercise Group and Control Group. Bonferroni-adjusted pairwise comparisons indicated that happiness scores significantly increased in the Swimming Exercise Group from pretest (*M* = 22.76, SD = 5.59) to posttest (*M* = 28.47, SD = 3.76), mean difference = 5.71, 95% CI (4.20, 7.22), *p* < 0.001. In contrast, no statistically significant change was observed in the Control Group (pretest: *M* = 25.71, SD = 6.01; posttest: *M* = 25.76, SD = 5.84), mean difference = 0.05, 95% CI (−1.46, 1.55), *p* = 0.949.

However, the main effect of the time factor was found to be statistically significant on the level of happiness [*F*(1, 40) = 29.84, *p* = 0.001, η^2^*p* = 0.42]. However, considering the significant time × group interaction, the main effect of time was evaluated contextually and not interpreted on its own. The main effect of group was found to be statistically insignificant [*F*(1, 40) = 0.06, *p* = 0.940, η^2^*p* = 0.01].

**Figure 2:**
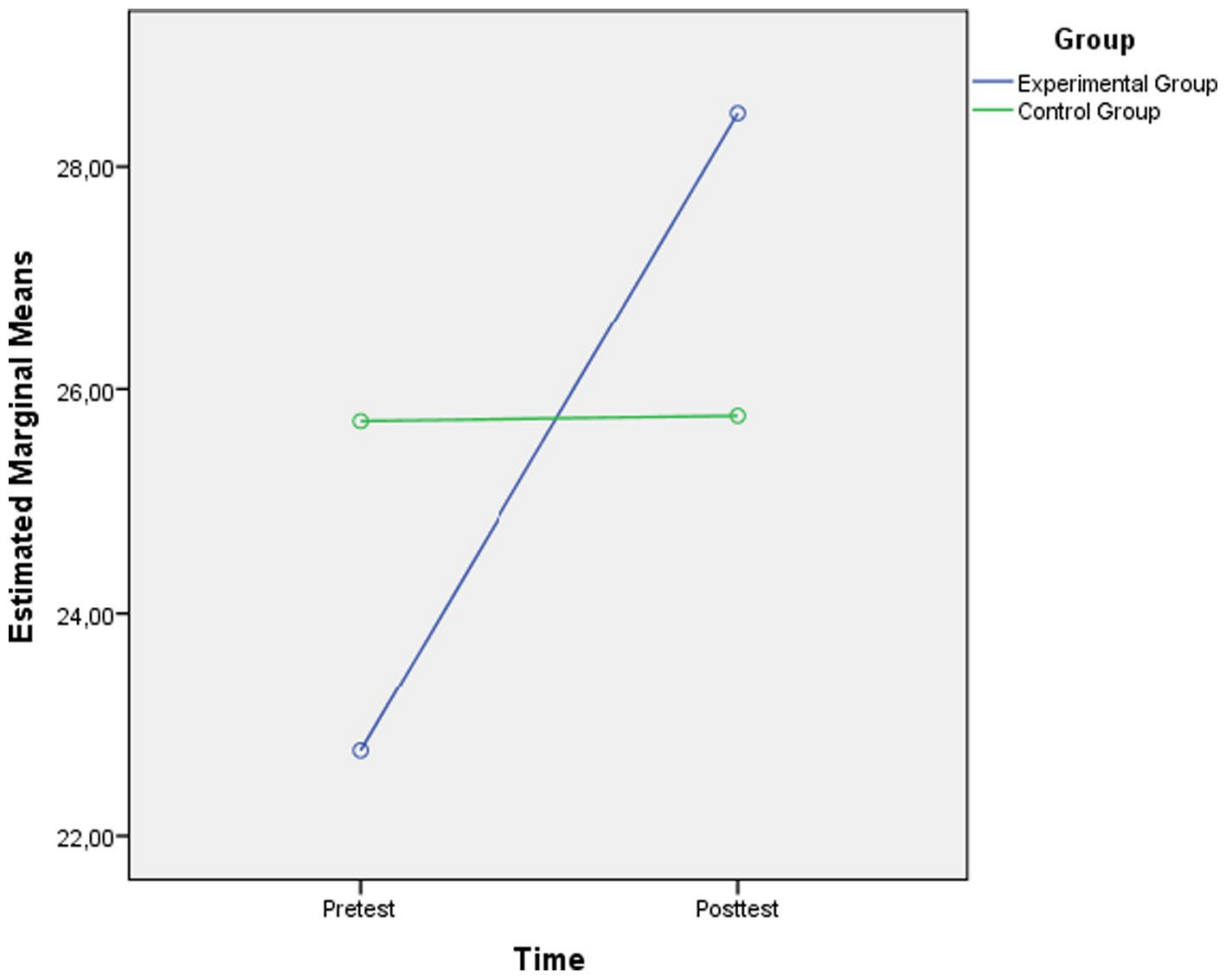
Changes in happiness scores from pretest to posttest in the Swimming Exercise Group (experimental group) and Control Group.

The graph showing the pretest and posttest mean scores of the Swimming Exercise Group and Control Group in terms of happiness level is presented in Figure 2.

As shown in Figure 2, the happiness level of the swimming exercise group increased significantly from the pretest to the posttest. In the control group, however, no change was observed between measurements.

## Discussion

The present study examined the effects of a 10-week structured swimming exercise program on hopelessness and happiness levels in female sport sciences students. The findings indicate that participation in regular, supervised swimming exercise was associated with significant reductions in hopelessness and significant increases in happiness, whereas no meaningful changes were observed in the Control Group. Taken together, these results suggest that structured swimming exercise may support emotional wellbeing within this non-clinical and physically active university subgroup.

The reduction in hopelessness observed in the Swimming Exercise Group is consistent with previous evidence indicating that regular physical activity may alleviate negative emotional states and improve future-oriented cognitive patterns (Craft and Perna, 2004; Rebar et al., 2015). Exercise participation has been linked to enhanced emotional regulation, improved stress tolerance, and increased perceived control, all of which may be conceptually relevant to hopelessness. In the present study, the significant time × group interaction and the moderate effect size (η^2^*p* = 0.30) indicate a statistically robust improvement in hopelessness within the intervention group. However, because the sample consisted of healthy female sport sciences students and the outcome was assessed through self-report measurement, this finding should be interpreted as a statistically significant improvement in perceived emotional status rather than as evidence of definitive clinical change.

Similarly, the increase in happiness in the Swimming Exercise Group is in line with literature demonstrating that structured physical activity can enhance positive affect and subjective wellbeing in young adults (Biddle et al., 2019; Huang et al., 2024). The large interaction effect (η^2^*p* = 0.41) suggests a substantial statistical effect within this sample. Nevertheless, in the absence of established clinical cut-off values or minimal clinically important difference (MCID) calculations, the observed increase in happiness should be interpreted as statistically and practically relevant within the context of the present study rather than as evidence of clinically established change or broadly generalizable clinical improvement. Accordingly, the present findings are better understood as favorable self-reported emotional changes associated with participation in a structured swimming exercise program.

An important implication of the findings is that emotional benefits were observed despite the participants being enrolled in a Faculty of Sport Sciences, where baseline familiarity with exercise and relatively high levels of physical activity might be expected. This suggests that the psychological contribution of a structured, progressive, and supervised aquatic exercise program may extend beyond routine physical activity exposure. In other words, the added value of the intervention may lie not only in exercise participation itself, but also in its regularity, supervision, progressive structure, and the potentially restorative characteristics of the aquatic environment.

At the same time, the findings should be interpreted within the characteristics of the study sample. Because the participants were female students enrolled in a sport sciences faculty, they likely represent a relatively homogeneous, physically active, and exercise-familiar subgroup. This characteristic may have influenced both their willingness to participate and their responsiveness to an exercise-based intervention. Therefore, although the present results are meaningful within this context, they should not be generalized to the broader university student population without caution.

Several limitations of the present study should be acknowledged. First, the sample consisted exclusively of female students enrolled in a Faculty of Sport Sciences at a single university, which limits the external validity of the findings. Because this group is likely to be more familiar with exercise and more positively oriented toward physical activity than many other student populations, the results should be interpreted within the context of a relatively homogeneous and physically active sample. Second, emotional outcomes were assessed exclusively through self-report measures, which may have increased susceptibility to response bias, expectancy effects, and demand characteristics, particularly because participant blinding was not feasible in the context of an exercise intervention. Accordingly, the observed effects should be interpreted as changes in self-reported emotional status rather than as objective psychophysiological outcomes. Third, physical activity in the Control Group was monitored only through weekly verbal self-report, and no objective method, such as accelerometry, activity logs, or a standardized physical activity questionnaire, was used. Therefore, uncontrolled variation in habitual physical activity cannot be completely ruled out. Finally, although the sample size was sufficient for the planned analyses, it remained relatively small, and the absence of long-term follow-up prevents any conclusion regarding the durability of the observed emotional changes over time.

Future research should include more diverse university populations, including students from different academic disciplines, male participants, and individuals with varying levels of baseline physical activity. Studies incorporating objective monitoring tools for physical activity, longer follow-up periods, and additional physiological or behavioral indicators of emotional regulation would help clarify the mechanisms underlying the observed effects and strengthen the external validity of the findings. Comparative studies examining aquatic and land-based exercise modalities may also provide a clearer understanding of whether swimming offers distinct emotional benefits in university settings.

## Conclusion

This study demonstrated that a 10-week structured swimming exercise program was associated with significant reductions in hopelessness and significant increases in happiness among female students enrolled in a Faculty of Sport Sciences. The observed moderate-to-large effect sizes suggest that structured swimming exercise may support self-reported emotional wellbeing in this specific university subgroup.

However, the findings should be interpreted cautiously. The sample was relatively homogeneous and consisted of physically active, exercise-familiar, non-clinical participants from a single academic setting. Therefore, the results should not be generalized to the broader university student population. In addition, the use of self-report measures and the absence of objective control-group activity monitoring further limit the strength of broader inferences.

Overall, swimming appears to be a potentially useful supportive exercise modality for emotional wellbeing in female sport sciences students, but further studies involving more diverse student populations, objective monitoring strategies, and longer follow-up periods are needed before broader conclusions can be drawn.

## Data Availability

The raw data supporting the conclusions of this article will be made available by the authors, without undue reservation.
